# Study protocol: EXERcise and Cognition In Sedentary adults with Early-ONset dementia (EXERCISE-ON)

**DOI:** 10.1186/1471-2377-12-75

**Published:** 2012-08-16

**Authors:** Astrid M Hooghiemstra, Laura HP Eggermont, Philip Scheltens, Wiesje M van der Flier, Jet Bakker, Mathieu HG de Greef, Peter A Koppe, Erik JA Scherder

**Affiliations:** 1 Department of Clinical Neuropsychology, VU University, Amsterdam, The Netherlands; 2 Alzheimer Centre, VU University Medical Centre, Amsterdam, The Netherlands; 3 Department of Epidemiology and Biostatistics, VU University Medical Center, Amsterdam, The Netherlands; 4 Department of Physiotherapy, Hospital Amstelland, Amstelveen, The Netherlands; 5 Department of Movement Sciences, Faculty of Behavioural and Social Sciences, University of Groningen, Groningen, the Netherlands; 6 Reade, location Hospital Amstelland, Amstelveen, the Netherlands

**Keywords:** Dementia, Early-onset dementia, Presenile dementia, Intervention, Physical activity, Exercise, Randomized controlled trial

## Abstract

**Background:**

Although the development of early-onset dementia is a radical and invalidating experience for both patient and family there are hardly any non-pharmacological studies that focus on this group of patients. One type of a non-pharmacological intervention that appears to have a beneficial effect on cognition in older persons without dementia and older persons at risk for dementia is exercise. In view of their younger age early-onset dementia patients may be well able to participate in an exercise program. The main aim of the EXERCISE-ON study is to assess whether exercise slows down the progressive course of the symptoms of dementia.

**Methods/Design:**

One hundred and fifty patients with early-onset dementia are recruited. After completion of the baseline measurements, participants living within a 50 kilometre radius to one of the rehabilitation centres are randomly assigned to either an *aerobic exercise program in a rehabilitation centre* or a *flexibility and relaxation program in a rehabilitation centre*. Both programs are applied three times a week during 3 months. Participants living outside the 50 kilometre radius are included in a feasibility study where participants join in a *daily physical activity program set at home making use of pedometers*. Measurements take place at baseline (entry of the study), after three months (end of the exercise program) and after six months (follow-up). Primary outcomes are cognitive functioning; psychomotor speed and executive functioning; (instrumental) activities of daily living, and quality of life. Secondary outcomes include physical, neuropsychological, and rest-activity rhythm measures.

**Discussion:**

The EXERCISE-ON study is the first study to offer exercise programs to patients with early-onset dementia. We expect this study to supply evidence regarding the effects of exercise on the symptoms of early-onset dementia, influencing quality of life.

**Trial registration:**

The present study is registered within The Netherlands National Trial Register (ref: NTR2124)

## Background

Early-onset dementia (EOD; < 66 years) is less common than late-onset dementia (LOD; > 65 years) [1]. In the majority of studies, Alzheimer’s disease (AD) is the most common subtype of EOD, followed by vascular dementia (VaD) and frontotemporal dementia (FTD) [2-4]. The clinical presentation of EOD, concerning cognitive and behavioural disturbances, is quite heterogeneous and depends on the neuropathological substrate [5-9]. EOD places a large psychological and economical burden on patients and caregivers because of the patients’ prominent role in society (having young children, working) at disease onset [10].

Despite the devastating impact of the disease, few intervention studies focus on this specific group of younger patients. Some pharmacological studies report the inclusion of (a small subset of) EOD patients [11-13]. Non-pharmacological intervention studies, such as exercise interventions studies, do not report to include EOD patients. Notably, there is an increasing number of studies examining the effects of exercise on cognitive and behavioural functioning in sedentary older people [14], in patients in a very early stage of LOD [15], and in patients in a moderate stage of LOD [16]. Most evidence of these studies points in the direction of beneficial effects of exercise on cognition in the aging population. Although, in view of their younger age many EOD patients may be better able to perform aerobic physical activity compared to healthy elders and LOD patients, certain dementia characteristics such as apathy may lead to sedentary and socially impoverished lifestyles [17-19]. Indeed, a ‘frontal’ presentation, i.e. symptoms as apathy and dysexecutive functioning, is relatively common in EOD patients [9].

A brain region that plays a pivotal role in executive functions is the prefrontal cortex [20]. Interestingly, especially those functions mediated by the prefrontal cortex react positively to increased physical activity [21]. The functioning of other cortical areas such as the parietal lobe also show a positive relationship with physical activity [22]. Involvement of the prefrontal and parietal cortices in exercise benefits stems form neuro-imaging studies in older adults that show greater task-related activity after an exercise intervention in regions of the prefrontal and parietal cortices during an executive function task [23]. It is noteworthy that particularly the prefrontal cortex and the parietal lobe are vulnerable in EOD [24,25] and may therefore offer a potentially appropriate venue for intervention. Furthermore, EOD patients are relatively young and suffer from less physical inconveniences compared to LOD patients [26] which makes participation in an intensive exercise program feasible and may result in larger benefits of the intervention [27].

The earlier mentioned positive effects of exercise combined with the characteristics of EOD patients, makes the lack of studies examining the effects of physical activity in the EOD population a remarkable observation. An elaborate description of the theory behind this observation is described in our review, also including a brief summary of part of the present study protocol [28]. In the present manuscript we present the detailed description of the entire protocol. This study is the first to investigate the exercise effects on cognition and behaviour in EOD patients. In the “*EXERcise and Cognition In Sedentary adults with Early‐ONset dementia study*” (EXERCISE-ON study), three different exercise programs are offered to persons with EOD: an aerobic exercise program in a rehabilitation centre, a flexibility and relaxation program in a rehabilitation centre and a daily physical activity program at home using pedometers (for a description see section *Interventions*). The present study is divided into two parts: a randomized controlled trial (RCT) and a feasibility study. The main aim of this study is to assess, making use of an RCT design, whether exercise slows down the progressive course of the symptoms of dementia, with respect to cognition, in particular psychomotor speed and executive functioning, and (instrumental) activities of daily living ((i)ADL), and may subsequently lead to better quality of life and less caregiver burden. In addition, a feasibility study is conducted to evaluate whether a physical activity program offered at home using pedometers can bring positive effects for EOD patients and their caregivers.

## Methods/Design

### Study design

**Figure 1:**
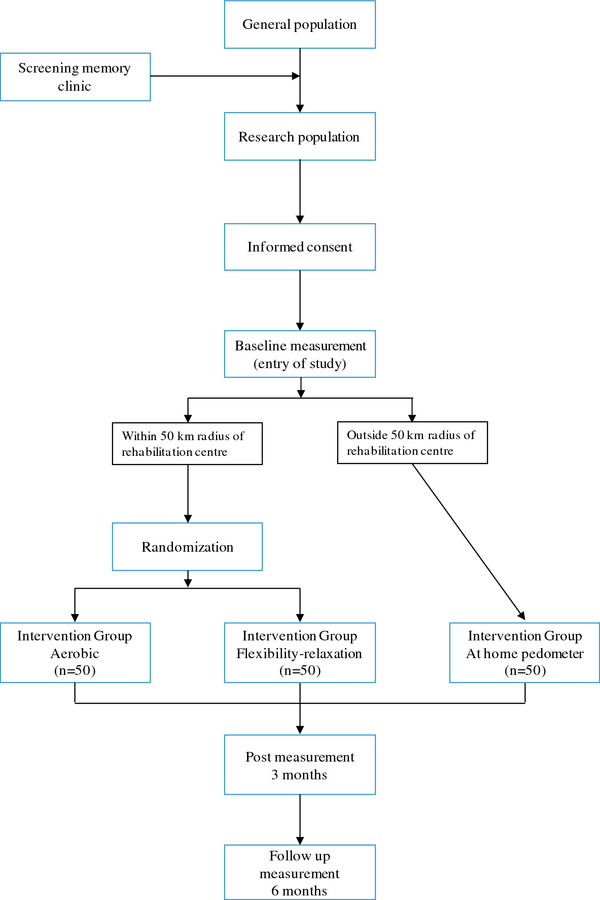
Trial design.

This study consists of two parts. The main study is designed as a randomized controlled trial with one hundred EOD patients. In addition a feasibility study will be conducted with fifty EOD patients (Figure 1).

### Participants

This study will include persons with a diagnosis of EOD: AD, VaD, FTD or other types of dementia. Participants will be recruited primarily in the Alzheimer centre of the VU University medical centre (VUmc) in Amsterdam, the Netherlands. Secondarily, participants will be recruited through affiliate memory clinics. A careful screening process, including medical history, physical, neurological, and neuropsychological examination as well as laboratory tests, electroencephalogram (EEG) and magnetic resonance imaging (MRI), will lead to the identification of patients with EOD. Diagnoses are made based on a multidisciplinary consensus team conference, according to the clinical criteria of the Diagnostic and Statistical Manual of Mental Disorders-IV-TR (DSM-IV-TR) [29], on the National Institute of Neurological Disorders and Stroke Alzheimer’s Disease and Related Disorders Association (NINCDS-ADRDA) for probable AD [30]; and on the National Institute of Neurological Disorders and Stroke-Association Internationale pour la Recherche et l'Enseignement en Neurosciences (NINDS-AIREN) for VaD [31]. Initially participants are recruited by a neurologist during a clinic visit where the patient, caregiver, and other family members are informed of the diagnosis. After provisional consent the study is explained (verbally and by use of printed material) by the principal investigator (AH), after which formal written consent is obtained.

*Inclusion criteria* are the following: 1) Diagnosis of EOD (onset of complaints < 66 years) (among others: AD, VaD, FTD); 2) Relatively early stage of dementia (Mini Mental State Examination (MMSE) score > 15 [32]); 3) Primary caregiver available. Participants will be *excluded* from participation when they 1) are wheelchair-bound; 2) are diagnosed with a neurodegenerative disease that primarily results in motor impairments, such as Parkinson's disease; 3) are diagnosed with serious cardiovascular disease, such as heart failure; 4) have a history of substance abuse; 5) had a head injury involving loss of consciousness greater than 15 minutes; 5) have a history of major psychiatric illness (e.g. personality disorder, schizophrenia); 6) have severe visual problems; 7) have severe hearing problems; 8) have insufficient proficiency of the Dutch language.

### Randomization

After obtaining written informed consent and completion of the baseline measurement, patients are assigned to one of the exercise programs. Originally, it was planned to randomize patients in one of three exercise programs, with an allocation ration of 1:1:1. However, during the pilot study travel distance to the rehabilitation centres appeared to be a restraint for many patients (even when patients were brought with taxis). The randomization procedure had to be adjusted. Patients who are living within 50 kilometres of one of the participating rehabilitation centres are randomly assigned to either the aerobic exercise program or the flexibility and relaxation exercise program. The allocation ratio is 1:1. To ensure the allocation ratio block randomization is used (block size is 4). Randomization is provided by e-mail. An anonymous list with identification numbers is sent to an independent researcher who is blinded to the identity of the patients. This independent researcher keeps the randomization list. Patients living outside a 50 kilometre radius of one of the rehabilitation centres are included in the daily physical activity program at home using pedometers.

### Interventions

Patients living within a radius of 50 kilometres of one of the rehabilitation centres are randomly assigned to one of the following programs:

#### Aerobic exercise program in a rehabilitation centre

This aerobic exercise program aims to improve the cardiorespiratory fitness of the participants. Activities consist of a warming-up, a core activity, i.e. cycling on a cycling ergometer, and a cooling down. The program endures three months, takes place three times a week, and is build up in duration and intensity [33]. Group size varies from 2 to 5 participants. This program is guided by a physical therapist (JB).

#### Flexibility and relaxation program in rehabilitation centre

The setting of this program is the same as for the aerobic exercise program (duration, frequency, group size, rehabilitation centre, and physical therapist). The difference is in the activities and the intensity of the program. Activities consist of stretching and toning exercises in combination with relaxation exercises. No build up is used.

Patients living outside the 50 kilometre radius from one of the rehabilitation centres are included in the following program:

#### Daily physical activity program at home using pedometers

This program takes place at the participant’s home. The program is based on the COACH method [34], developed by the interfaculty Centre for Human Movement Sciences of the University of Groningen (Netherlands) and the Centre for Movement and Research (CBO), Groningen (Netherlands). The COACH method is an evidence-based method to stimulate sedentary individuals to enhance physical activity participation in daily life, using “exercise counselling”. The exercise counselling is focused on intrinsic motivation [35], since this type of motivation is predominantly related to sedentariness [36] and is often disturbed in patients with brain damage [37]. Patients develop a benchmark for physical activity in several phases. Using 3 interviews, following motivational interviewing [38] and goal setting techniques [39,40], the participant’s attitude towards physical activity is discussed and changed if necessary. The interviews are given by a neuropsychologist (in training).

### Setting

Both the aerobic exercise program and the flexibility and relaxation program are given in two rehabilitation centres in the Netherlands (i.e. Reade, hospital Amstelland, Amstelveen; department of rehabilitation, Jeroen Bosch hospital,’s Hertogenbosch). The third exercise program (pedometer) is offered nation-wide at the patients’ homes.

### Measurements and procedures

Each participant will undergo three measurements: baseline measurement (entry of the study, prior to randomization), post measurement (end of the exercise program, three months after baseline measurement), and follow-up measurement (six months after baseline measurement). A measurement consists of a neuropsychological examination during approximately 2 hours. Thoroughly trained master students Clinical Neuropsychology, blinded to group allocation will administer the tests. In the week following the neuropsychological examination the participants wear a pedometer and an actiwatch activity monitor for one week. Participants that are able to visit one of the rehabilitation centres also receive a physical examination during 30 minutes*.*

### Primary outcome measures

1. Cognitive functioning

*Alzheimer’s Disease Assessment Scale (ADAS)-COG*[41]. The ADAS-COG is designed to evaluate cognitive disorders in persons with AD. The ADAS-COG is often used in clinical trials for dementia [42,43].

2. Psychomotor speed/executive functioning

*Trail Making Test* (TMT) [44]. The TMT consists of two parts. Part A gives a measure for psychomotor speed. The more complex Part B gives an indication of cognitive flexibility, which is a part of executive functioning.

3. (Instrumental) activities of daily living ((i)ADL)

*Disability Assessment for Dementia* (DAD) [45]. To gain insight in the (i)ADL of participants, the DAD questionnaire is administered. The DAD is an informant-report questionnaire specifically designed for persons with a (beginning) dementia. The DAD is administered to the caregiver and provides information with respect to how the patient performs (i)ADL’s and whether he/she is taking initiative to do things.

4. Quality of life

The *Dementia-Quality of Life* (D-QOL) [46] is a valid instrument to assess the quality of life in persons with a mild to moderate stage of dementia [47]. The D-QOL is a self-report questionnaire and consists of propositions in 5 subscales: self-esteem, positive effect, negative effect, feelings of belonging, and enjoying the environment.

### Secondary outcome measures

#### Physical measures

Physical fitness is assessed using the *Åstrand cycle test*[48], which is a submaximal measure of fitness and is used to estimate the maximal oxygen uptake (VO2max). Walking speed is measured using the *6 Minutes Walk Test*[49]. Balance and strength of the lower extremities is measured by the *Sit to Stand Test*[50]. Finally, the level of physical activity (steps/day) is assessed using a *pedometer* (OMRON HJ-113) [51,52]. Participants are also asked to report their level of physical activity during the last week. This is assessed using a self-report questionnaire, the *Physical Activity Scale for the Elderly (PASE)*[53].

#### Neuropsychological measures

An extensive neuropsychological battery is administered. Regarding episodic memory the *“Face Recognition”* from the *Rivermead Behavioural Memory Test (RBMT)*[54] is used to measure face recognition. Short term memory is measured using the *“Digit span forwards”,* working memory using the *“Digit span backwards”* from the *Wechsler Adult Intelligence Scale – Revised (WAIS-R)*[55]; Word fluency is assessed using *“category fluency” (animals and occupations)* from the *Groninger Intelligence Test (GIT)*[56]; The *Stroop Colour Word Test*[57] short version is used to measure interference and inhibition. Coding, psychomotor-, and processing speed is measured using the subtest *“Symbol substitution”* from the *(WAIS-R)*[55,58]. Furthermore, visuospatial capacity is assessed by the *“Perceptual closure”* subtest from the GIT [56]. Finally, self-efficacy is measured using a self-report questionnaire: *General Self-efficacy scale (GES):* Dutch version [59].

#### Rest-activity rhythm

The rest-activity rhythm is assessed using the Actiwatch activity monitor; Cambridge Neurotechnology Ltd., Cambridge, Great Britain [60].

### Demographic and control variables

At baseline, demographic information, i.e. age, sex, educational attainment (using the system of Verhage, ranging from 1 (low) to 7 (high) [61]), diagnosis of dementia, number of years since diagnosis, and currently prescribed medications will be collected. To determine whether there has been a change in subjective physical functioning and in the amount of encouragement needed to start exercising several questions are asked during recruitment. Outcome measures will also be controlled for co-morbid medical conditions (medical chart), depressive symptoms: *Centre for Epidemiologic Studies Depression Scale* (CES-D) [62-64], and Apolipoprotein E (ApoE) genotype [65,66], in view of possible moderating effects on treatment outcome.

### Statistical analysis

In the statistical analysis an intention-to-treat analysis will be performed in order to minimize bias [67]. Differences in the outcome measures between the aerobic exercise program and the flexibility and relaxation program on the three measurement moments will be analysed using a Linear Mixed Model (LMM) with contrasts. Time and treatment condition will be considered as a within-subjects and a between-subjects variable respectively. To analyse possible effects of the daily physical activity program at home using pedometers also a LMM is used, with time as within-subjects variable. Post-hoc analyses will be conducted to differentiate between persons who were physically active and persons who were physically inactive, based on pedometer outcomes on baseline measurement.

### Sample size

The sample size calculation (using G*power [68]) is based upon two meta-analyses concerning studies in which the effects of physical training on cognition was investigated in either older adults with a cognitive disorder or dementia [69] or in healthy sedentary older adults [14]. Summary effect sizes in these studies were respectively Hedges’ *g* = 0.57 and *g* = 0.68, which are considered medium effect sizes. Since in the RCT part of the present study the control group receives an intervention existing of flexibility and relaxation exercises, in contrast to a control group who does not perform any physical activity, we assume a small effect size instead of a medium effect size (*f* = .1). For 80 % power, the sample size requirement (using a 5 % significance level) is 82 persons in total. Accounting for participants who withdraw from the study, as is seen in other intervention studies with healthy older adults [70] and patients with dementia [16] the total study population is targeted at 100 participants.

In the feasibility study a small to medium effect size was used (*f* = .15), in order to avoid Type II errors. Keeping the parameters equal to the situation above, the estimated sample size is 38 persons. Accounting for participants who withdraw from the study, the study population for this part of the study is targeted at 50 persons.

### Ethical and legal considerations

The protocol is reviewed and approved by the Extramural Medicine institute (EMGO) which is part of the Dutch School of Primary Care Research (CaRe) (ref: WC2009-046). Ethical aspects of the protocol were approved by the accredited Medical Review Ethics Committee (aMREC) of the VUmc (ref: 2009–220) according to the Declaration of Helsinki (2008). The research has been included in the general assessment and registration form (ABR form) (ref: NL27426.029.09) and in The Netherlands National Trial Register (NTR) (ref: NTR2124).

## Discussion

The EXERCISE-ON study will evaluate whether exercise slows down the progressive course of the symptoms of dementia in EOD patients. Characteristics of EOD patients, such as apathy, loss of initiative, together with their age and physique, make them good candidates for a physical activity program.

This study has several strengths. Despite the devastating impact of EOD on the lives of patients and their families, few specific treatments are available for EOD patients. First of all, EOD patients are dependent on facilities developed primarily for elderly, and second of all, EOD patients are underrepresented in scientific studies. Some pharmacological studies report the inclusion of (a small subset of) EOD patients. To our knowledge the present study is the first study that offers exercise interventions to EOD patients. Another strength is that the exercise programs are offered in different settings. This is the first study that brings patients suffering from dementia into a rehabilitation setting involving the guidance of an experienced physical therapist. In the rehabilitation centres patients are exposed to fellow EOD patients. Fellow patients can share experiences and tips, which may help getting grip on the consequences of a chronic disease [71]. The daily activity program is offered at participants homes. Particular this setting stimulates potential implementation in the future.

A challenge in this study is the inclusion of a sufficient number of participants. EOD is a rare condition, the proportion of people with EOD varies between 7.3 % to 31 % in studies from Japan, the UK, and the USA [4,10,72,73]. Furthermore, it is expected that EOD patients have busier lives and are involved in more activities in contrast to patients suffering from dementia at an older age, similar to healthy adults of middle and older age [74]. To overcome this challenge we include multiple rehabilitation centres and also offer a home-based daily physical activity program to patients that are not able to travel to one of the rehabilitation centres.

In summary, the EXERCISE-ON study is an innovative study examining possible beneficial effects of exercise on symptoms, with respect to cognition, (i)ADL, and quality of life, of EOD. Study results may contribute substantially to care facilities for EOD patients. Furthermore, exercise may offer a new set of coping skills for this patient group.

## Abbreviations

EXERCISE-ON, EXERcise and Cognition In Sedentary adults with Early-ONset dementia; EOD, Early-Onset Dementia; LOD, Late-Onset Dementia; AD, Alzheimer’s Disease; VaD, Vascular Dementia; FTD, Frontotemporal Dementia; RCT, Randomized Controlled Trial; (i)ADL, (instrumental) Activities of Daily Living; VUmc, VU University medical centre; EEG, Electroencephalogram; MRI, Magnetic Resonance Imaging; DSM-IV-TR, Diagnostic and Statistical Manual of Mental Disorders-IV-Text Revision; NINCDS-ADRDA, National Institute of Neurological and Communicative Disorders and Stroke and the Alzheimer's Disease and Related Disorders Association; NINDS-AIREN, National Institute of Neurological Disorders and Stroke and the Association Internationale pour la Recherche et l'Enseignement en Neurosciences; MMSE, Mini Mental State Examination; CBO: “Centrum voor Beweging en Onderzoek” (Dutch), Centre for Movement and Research; ADAS-COG, Alzheimer’s Disease Assessment Scale-cognition; TMT, Trail Making Test; DAD, Disability Assessment for Dementia; D-QOL, Dementia-Quality of Life; VO2max, Maximal Oxygen uptake; PASE, Physical Activity Scale for the Elderly; RBMT, Rivermead Behavioural Memory Test; WAIS-R, Wechsler Adult Intelligence Scale-Revised; GIT, Groninger Intelligence Test; GES, General Self-efficacy Scale; CES-D, Center for Epidemiologic Studies Depression scale; ApoE, Apolipoprotein E; LMM, Linear Mixed Model; EMGO, Extramural Medicine institute; CaRe, Dutch School of Primary Care Research; aMREC, Accredited Medical Ethical Committee; ABR form, General assessment and registration form; NTR, The Netherlands National Trial Register.

## Competing interests

The authors declare that they have no competing interests.

## Authors’ contributions

ES, LE, and PK conceived the idea of this study. ES and LE wrote the grant application of the study. AH, LE and ES developed the intervention programs and the protocol of outcome measures. AH coordinates the study under direct supervision of LE and ES. PS and WMF enable the recruitment and selection of EOD patients in the Alzheimer Center of the VUmc and have an advisory role in the project. PK screens the participants on motor disturbances before participation. JB executes the exercise interventions. MG supplied the daily physical activity program at home using pedometers and trained AH in performing the interviews. AH was the primary author for this manuscript. LE and ES helped draft this manuscript. All authors provided critical feedback and approved the final manuscript.

## Pre-publication history

The pre-publication history for this paper can be accessed here:

http://www.biomedcentral.com/1471-2377/12/75/prepub
